# Disregard of neurological impairments associated with neglected tropical diseases in Africa

**DOI:** 10.1016/j.ensci.2015.11.002

**Published:** 2015-11-07

**Authors:** Emmanuel Quansah, Esther Sarpong, Thomas K. Karikari

**Affiliations:** a Pharmacology, School of Health and Life Sciences, De Montfort University, Leicester LE1 9BH, UK; b Department of Molecular Biology and Biotechnology, School of Biological Science, University of Cape Coast, Cape Coast, Ghana; c Neuroscience, School of Life Sciences, University of Warwick, Coventry CV4 7AL, UK; d Midlands Integrative Biosciences Training Partnership, University of Warwick, Coventry CV4 7AL, UK

**Keywords:** DALYs, disability adjusted life-years, DRC, Democratic Republic of Congo, GBD, Global Burden of Disease, NTDs, neglected tropical diseases, WHO, World Health Organization, Neglected tropical diseases, Neurological impairments, Infectious diseases, Africa, Poverty

## Abstract

Neglected tropical diseases (NTDs) affect people in the bottom billion poorest in the world. These diseases are concentrated in rural areas, conflict zones and urban slums in Africa and other tropical areas. While the World Health Organization recognizes seventeen priority NTDs, the list of conditions present in Africa and elsewhere that are eligible to be classified as NTDs is much longer. Although NTDs are generally marginalized, their associated neurological burden has been almost completely disregarded. However, reports indicate that trichuriasis, schistosomiasis and hookworm infection, among others, cause impairments in memory and cognition, negatively affecting school attendance rates and educational performance particularly among children, as well as agricultural productivity among adults. Consequently, the neurological impairments have substantial influence on education and economic productivity, thus aggravating and perpetuating poverty in affected societies. However, inadequate research, policy and public health attention has been paid to the neurological burdens associated with NTDs. In order to appropriately address these burdens, we recommend the development of policy interventions that focus on the following areas: (i) the introduction of training programs to develop the capacity of scientists and clinicians in research, diagnostic and treatment approaches (ii) the establishment of competitive research grant schemes to fund cutting-edge research into these neurological impairments, and (iii) the development of public health interventions to improve community awareness of the NTD-associated neurological problems, possibly enhancing disease prevention and expediting treatment.

## Highlights

• Neglected tropical diseases (NTDs) disproportionately affect the world’s poorest populations
• While NTDs are generally marginalized, their associated neurological burden is almost completely disregarded in Africa
• Neurological impairments of NTDs disrupt cognition and memory, thus adversely impacting education and health of affected families.
• Policy interventions focused on advancing clinical research and early diagnosis and treatment paradigms are encouraged.

## 1 Introduction

Neglected tropical diseases (NTDs) are a set of predominantly infectious diseases that are particularly prevalent in resource-poor populations, mostly in tropical areas. The World Health Organization (WHO) recognizes seventeen NTDs that disproportionately affect the world's poorest populations [1]. The WHO estimates that more than 1 billion of the world's poorest 2.7 billion people (living on less than US$ 2.00 a day) are affected by one or more NTDs [1]. These diseases do not only spread in poverty-stricken areas, but they also aggravate and perpetuate the poverty of affected societies [2], [3]. NTDs cause considerable disfigurement, morbidity and mortality. Some of these diseases impair childhood growth and development, decrease economic productivity of young adults, and cause adverse pregnancy outcomes among infected individuals [4]. NTDs can be controlled and, in some cases, eradicated using low-cost strategic interventions [1]. However, although some local and international organizations have actively been contributing to the prevention and effective treatment of these illnesses, the impact has been less compared to diseases such as malaria, HIV/AIDS and tuberculosis [1]. A probable reason for the seeming neglect of NTDs is that the recorded mortality resulting from these diseases is low compared to mortality caused by diseases such as HIV/AIDS and malaria [5]. However, authorities should be cognizant of the fact that NTDs and their related burdens are often under-reported since the diseases are common in rural areas that are not adequately covered by health surveillance systems [4].

Several NTDs have known neurologic impairments (e.g. cysticercosis, leprosy, dengue, and rabies). However, the neurologic aspects of these NTDs are given much less attention. Nonetheless, addressing the neurologic aspects of NTDs would be particularly important in supporting vulnerable populations and the establishment of the true health and socio-economic burdens of these diseases [6]. Here, we highlight the seemingly unrecognized burden of neurologic complications associated with NTDs, and advocate for improvements in the policy, research and public health attention paid to them.

## 2 NTD-related neurology: a burden requiring urgent attention in Africa

NTDs are given only limited attention by the public health community at both the national and international levels [3]. Most of these diseases are communicable, and may be classified by aggregating them according to their causative agents (viral, bacterial, protozoan etc.), biology (infectious or noninfectious) and clinical manifestations (primarily neurological, or not). NTDs like Konzo (prevalent in the Kahemba territory of the Democratic Republic of Congo (DRC) and other countries such as Mozambique, Cameroon, Tanzania, Central African Republic and Angola) that result from food poisoning are classified among the non-infectious disease group [7], while most others (listed in Table 1), are infectious in nature. The NTDs listed in Table 1 are endemic in, but not limited to, specific African countries. In particular, hookworm infections and schistosomiasis cause substantial neurological impairments and represent a significant burden in affected communities [5], [8]. It is estimated that about 600–700 million people suffer from hookworm infections globally, with most of the infections occurring in Nigeria, the DRC and some Asian countries [5], [8]. Remarkably, about 90% of the 207 million global cases of schistosomiasis occurs in sub-Saharan Africa, especially in Nigeria, Ghana, Tanzania and the DRC [9].

**Table 1:** Neurologic impairments associated with neglected tropical diseases (NTDs) endemic in Africa.

Type of disease by cause	NTDs	Associated neurologic impairments
Protozoan infection	Human African trypanosomiasis*, leishmaniasis*	Neural axis affected: central, peripheral and autonomic nervous systems Suggested impairments: neuropathy, myelopathy, meningo-encephalitis, and stroke, as well as death in severe cases
Bacterial infection	Leprosy*, buruli ulcer*, trachoma*,	
Viral infection	Rabies, dengue fever	
Helminth infection	Taeniasis/cysticercosis, schistosomiasis*, soil transmitted helminthiasis (ascariasis*, hookworm infection*, trichuriasis*), lymphatic filariasis*, onchocerciasis*, dracunculiasis*, foodborne trematodiasis: fascioliasis, paragonimiasis	

NTDs, neglected tropical diseases; *twelve core NTDs.

Some international organizations have attempted to estimate the burden of NTDs globally. Data from the WHO's Global Burden of Disease (GBD) project indicate that a group of twelve NTDs (onchocerciasis, lymphatic filariasis, dengue, leprosy, leishmaniasis, schistosomiasis, human African trypanosomiasis, Japanese encephalitis, Chagas disease, and intestinal nematode infections such as trichuriasis, hookworm disease and ascariasis) accounted for about 177,000 deaths globally in 2002, and that these deaths mostly occurred in sub-Saharan Africa [10]. According to the WHO's GBD estimate, about 20 million disability adjusted life-years (DALYs) were lost due to these NTDs [10]. Recently-revised estimates, however, include a slightly different set of twelve NTDs (leprosy, leishmaniasis, human African trypanosomiasis, schistosomiasis, hookworm infection, trichuriasis, ascariasis, onchocerciasis, dracunculiasis, lymphatic filariasis, trachoma, and Chagas disease) that accounted for an even higher burden, including 56.6 million DALYs and 534,000 deaths [11]. Although NTDs have been given limited attention in comparison with malaria or HIV/AIDS, when the chronic morbidities of NTDs (particularly hookworm infection and schistosomiasis) are fully considered, they rank among the most important diseases in Africa [5]. Overall, NTDs account for 4.5–92 million DALYs annually, and the upper limit is greater than the DALYs resulting from HIV/AIDS or malaria [5]. Additionally, conservative estimates for food borne trematode infections (paragonimiasis, opisthorchiasis, fascioliasis, clonorchiasis, and intestinal fluke infections) alone caused an additional 665,352 DALYs and 7158 deaths [12].

These data indicate that the endemicity of NTDs in sub-Saharan Africa accounts for a considerable socio-economic burden. Surprisingly, the available disease-burden estimates do not take into account the NTD-associated neurological problems. Therefore, the actual burden of neurological problems arising from NTDs in Africa largely remains unknown.

## 3 Challenges in assessing the burden of neurological impairments associated with NTDs in Africa

There are several inherent challenges involved in assessing the true burden of neurological impairments of NTDs. These challenges, among other things, do account for the under-appreciation and seeming ‘neglect’ of NTDs. The challenges include the following: (i) individuals suffering from NTD-associated neurological impairments are usually part of the poorest groups in their societies (possibly living in remote, impoverished communities) and may therefore not be adequately covered by national health interventions (ii) data utilized in disease assessment schemes are largely based on hospital records (owing to epidemiological challenges in many African communities) and are therefore limited to only those who get access to medical facilities. However, many people in rural Africa lack access to hospital facilities. (iii) the full disability and morbidity associated with a given NTD may be rather difficult to evaluate, as a ‘syndromic’ approach is often employed in assessing morbidity rather than a ‘disease-specific’ approach. Consequently, only striking symptoms are taken into account while the so-called ‘subtle morbidity’ is often not considered. (iv) finally, attributing the cause of death to a particular disease may be both challenging and inaccurate because co-infections are common i.e. individuals are often affected by more than one lethal condition (e.g. parasitic diseases such as hookworm or schistosomiasis complicate HIV/AIDS and tuberculosis) [4].

## 4 Why further attention should be paid to neurological impairments associated with NTDs

NTDs represent a potent reinforcement of poverty due to the huge socio-economic burden they present to endemic communities (Table 2). These burdens affect education, child health and survival, as well as economic and agricultural productivity. The bacterial and parasitic (protozoan and helminthic) tropical infections and dengue are collectively responsible for the highest burden of NTDs [5]. The soil-transmitted helminth infections (trichuriasis, hookworm, ascariasis), schistosomiasis, lymphatic filariasis, onchocerciasis (river blindness) and trachoma are also tropical diseases with high prevalence rates but are quite amenable to control [13]. About 600–800 million people, mostly children, are known to suffer from soil-transmitted helminthic infections [13]. Among these, hookworm infection, which causes maternal and childhood anemia, results in the greatest disability, and represents one of the highest burden of NTDs in Africa [8], [13]. The neurologic impairments associated with schistosomes and soil-transmitted helminthes lead to detrimental effects on education, school attendance and performance, and future job prospects and earnings [14], [15]. These effects are, at least in part, due to impairments in memory and cognition, as previously reported in individuals with chronic hookworm infection, schistosomiasis, and trichuriasis [14], [15]. However, deworming programs have demonstrated that combating NTDs in childhood represent a cost-effective approach for improving education in endemic communities [16]. Investing as little as US$3.50 per child on disease control could lead to a gain of an extra school year [16].

**Table 2:** Socio-economic burden associated with different categories of neglected tropical diseases (NTDs).

NTDs	Associated socio-economic burden
Hookworm infection; schistosomiasis; onchocerciasis; trichuriasis; ascariasis	Adverse effects on education
Onchocerciasis; trachoma; schistosomiasis; hookworm infection; dracunculiasis; lymphatic filariasis	Decreased agricultural productivity
Leishmaniasis; onchocerciasis; human African trypanosomiasis; lymphatic filariasis	Disease treatment-related economic burden
Schistosomiasis; hookworm infection; trichuriasis; ascariasis	Reduced child survival

NTDs, neglected tropical diseases.

Investing in disease control could rescue hard-hit communities focusing on four key sectors: agriculture, infrastructure, education and health (particularly maternal and child health) [5]. Since NTDs mostly affect families in rural areas depending mostly on subsistence agriculture, the most direct impacts may include impaired worker productivity and reduced harvests. Lymphatic filariasis or anemia arising from schistosomiasis and hookworm infection, and blindness resulting from onchocerciasis and trachoma can lead to both short- and long-term substantial reduction in agricultural productivity among the affected individuals, sometimes preventing these individuals from working again [17]. The major NTDs also have adverse economic consequences on affected families through healthcare costs. Schistosomiasis and the soil-transmitted helminth infections impair the growth, mental health and physical fitness of children [13]. A study involving 432 children aged 8–13 years showed that children with hookworm infection performed poorly in cognitive and motor tests than those without hookworm infection, suggesting that hookworm infection has a significant adverse influence on a child's working memory and may therefore impact reasoning ability and reading comprehension [14]. Moreover, by causing severe anemia during pregnancy, schistosomiasis and hookworm infection reduce child survival, contributing to maternal and infant mortality [18]. In addition, anemia resulting from hookworm infection can aggravate the clinical course of falciparum malaria, particularly in women and children [18]. These examples demonstrate that disease co-infection can exacerbate their associated problems.

Overall, NTDs represent a significant socio-economic and neurological burden on poor families in endemic rural communities and their high prevalence rate may prevent the achievement of the first six Millennium Development Goals [13]. Thus, developing cost-effective interventions aimed at disease control could improve the neurological health of affected individuals and prevent the disease-associated economic and social challenges.

## 5 Strategies for treating NTDs and their neurological complications

Most NTD-associated neurological problems are caused by infectious agents [1], [4]. Thus, almost all infectious NTDs with associated neurological impairments are not only preventable but also treatable, as chemotherapeutic agents and vaccines are available to neutralize the underlying pathogens [1], [5]. It must be noted, however, that the clinical picture is a little more complex as currently available medications usually only neutralize or kill the infectious agents before they cause illness, but often cannot curb the associated morbidity once it is established [4]. Therefore, if treatment could be made available without delay, the underlying infection could be stopped in time and its neurological consequences effectively contained. This clearly spells out a need for a public health strategy to effectively tackle the burden of neurological impairments associated with NTDs. A good strategy would be to emphasize primary and secondary prevention while not neglecting the tertiary preventive approach.

Primary prevention focuses on the underlying infection and employs measures aimed at preventing the establishment of infection in humans and reducing environmental transmission of the infection [4]. Examples include vector control measures against cycticercosis/taeniasis, *Trypanosoma brucei rhodesiense* in human African trypanosomiasis, or against the transmission cycle of rabies [4]. Provision of safe drinking water and improved public education on the benefits of improved sanitation on health are also likely to contribute immensely to risk reduction. In NTD-endemic areas, a secondary preventive measure targeted against the morbidity associated with already established infection is preferred. A timely administration of treatment at the initial stages of infection could prevent most disease-associated morbidity [19]. The WHO recommends preventive chemotherapy for the control of helminth infections in endemic areas [1]. Preventive chemotherapy involves large-scale distribution of drugs, in this case, anti-helminthic drugs at regular intervals to populations in endemic areas without individual diagnosis [1]. Such an approach keeps the number of infecting worms at the lowest levels possible, in spite of re-infection, hence controlling morbidity [1]. Tertiary prevention, on the other hand, is directed against disability resulting from established and advanced morbidity. Management of these patients normally involves palliative remedies. For instance, major neurological sequelae in individuals with human African trypanosomiasis remain even after treatment, disrupting the mental development of children and causing severe motor and psychiatric impairments in patients [4]. Rehabilitation and supportive therapies become the only options for patients with such advanced conditions [4].

The fact that early disease treatment can avert the establishment of potential neurological complications underscores the need to develop effective public awareness programs. Existing public health programs have mainly been led by international donor agencies [5], [19], [20]. Global financing mechanisms for the control of these tropical diseases must recognize, however, that for disease-control programs to be effective in Africa, they must be nationally owned, backed by political commitment and embedded in national health plans [5]. For example, community-directed interventions done through the African Program for Onchocerciasis Control (which included 31 endemic African countries as of 2012) have particularly been a success story, even in countries where only few other health programs have been successful [5], [19], [20]. Therefore, NTD interventions in Africa would need to maintain a community involvement and become a part of the overall health services scheme in NTDs prone African countries.

In addition, the low availability of published research findings on the neurological aspects of NTDs suggests that research interest in this area is low among African scientists. This may stem from the non-availability of training programs and funding schemes focused on this topic. We suggest the development of collaborative efforts between researchers working on NTDs and their colleagues in the neurosciences to conduct further research into NTD-associated neurological problems. To do this, there may be the need to introduce programs such as workshops and short courses aimed at training more scientists and clinicians in tools and techniques that may strengthen their capacity in NTD-related research, diagnostic and treatment approaches. Furthermore, the establishment of competitive research grants schemes to fund cutting-edge research into NTD-related neurological impairments would also be of immense benefit.

## 6 Conclusion

NTDs represent a significant burden on poor families. Unfortunately, the disease-associated neurological problems have been given limited attention by the public health, political and research communities at both the national and international levels. Neurologic complications associated with schistosomes and soil-transmitted helminthes as well as other NTDs result in adverse effects on education, productivity, and wellbeing. These effects are attributable to impairments in memory and cognition, as reported in individuals with chronic hookworm infection, schistosomiasis, and trichuriasis. Increasing public awareness of the neurologic aspects of NTDs, dedicating further research and funding attention, and training more clinicians and scientists on appropriate research methods, clinical manifestations, diagnosis and treatment are some of the approaches that may help to improve early disease detection and treatment, and eventual eradication.

## Conflict of interests

The authors declare that there is no conflict of interest regarding the publication of this paper.
